# Evaluation and selection of alfalfa genotypes for tolerance to aluminium toxic stress

**DOI:** 10.3389/fpls.2024.1437993

**Published:** 2024-07-24

**Authors:** Aurelija Liatukienė, Regina Skuodienė, Eglė Norkevičienė, Sirje Tamm, Priit Pechter, Giedrius Petrauskas

**Affiliations:** ^1^ Lithuanian Research Centre for Agriculture and Forestry, Institute of Agriculture, Instituto al. 1, Kėdainiai dist., Lithuania; ^2^ Lithuanian Research Centre for Agriculture and Forestry, Vėžaičiai Branch, Klaipėda dist., Lithuania; ^3^ The Centre of Estonian Rural Research and Knowledge, J. Aamissepa 1, Jogeva, Estonia

**Keywords:** agro-biological traits, al in soil solution, cluster analysis, evaluation, selection, *Medicago sativa L*

## Abstract

Alfalfa is one of the most important and the most cultivated crop due to its high nutritive quality and yield, but adaptation of alfalfa genotypes differ in terms of mobile aluminium stress in the soil. The aim of this study was to evaluate the tolerance to mobile Al concentrations in the laboratory and in the naturally acidic soil and select the promising genotypes based on agro-biological traits. In 2019, a laboratory experiment was conducted at the Institute of Agriculture of LAMMC. The experiment in the acidic soil with different mobile Al concentrations was conducted at the Vėžaičiai Branch of LAMMC. In 2020, the crops of alfalfa genotypes (11 cultivars and 3 populations) were established on *Balthygleyic Dystric Retisol*. The agro-biological traits were assessed during the 2021–2022 season. The tolerance index of hypocotyls and roots was evaluated using the filter-based screening method at different AlCl_3_ (0.0–64 mM) concentrations. The study results of the filter-based screening method showed that the genotype Žydrūnė, Malvina, Jõgeva 118, Skriveru, and 3130 were the most tolerant ones and the hypocotyl tolerance index of these genotypes was higher compared to medium tolerant genotypes Birutė, PGR12489, Europe and AJ2024 at 8, 16, 32 and 64 mM AlCl_3_ concentrations. The hypocotyl and root tolerance index of medium tolerant genotypes was higher compared to a sensitive genotype PGR10249 at 8 and 16 mM AlCl_3_. The study of cluster analysis with mobile Al 0.0–65.0 mg kg^-1^ showed that the genotypes Žydrūnė, Europe, AJ2024 and 3130 were the best in terms of wintering and spring regrowth, the cultivar Malvina had the best value of wintering, height before flowering and stem number, the cultivar Birutė had the best value of spring regrowth, height before flowering and seed yield, and the cultivar Skriveru had the best value of spring regrowth, height before flowering, stem number and seed yield.

## 1 Introduction

Alfalfa (*Medicago sativa* L.) is one of the most important, oldest forage species and the most cultivated one due of its high nutritive quality, yield, and adaptation to various environmental and soil conditions (Li et al., 2007; Annicchiarico et al., 2015; Wenxu et al., 2019; Feng et al., 2022). Alfalfa is distinguished for a wide genetic variation in terms of adaptation to specific growth conditions, however, it is very strongly affected by soil acidity and aluminium (Al) toxicity (Annicchiarico and Pagnotta, 2012; Khu et al., 2012; Yang et al., 2013). The soil acidity increases (pH decreases), when the concentrations of mobile aluminium and hydrogen cations in the soil increase while base cations such as calcium, magnesium, potassium and sodium are leached out of the soil (Uexküll and Mutert, 1995; Gupta et al., 2013; Agegnehu et al., 2021). The crops have sensitive micromolar concentrations of mobile Al because mobile Al interferes with various physiological and cellular processes in the roots. High concentrations of mobile Al are toxic to plants and cause oxidative stress and may disrupt metabolic functions of plants (Langer et al., 2009). Normally, at low soil pH, mobile Al inhibits the growth of root tip cells and root elongation, resulting in slower root growth and reduced water and nutrient uptake (Mugai et al., 2000; Rangel et al., 2007; Yang et al., 2011, 2013; Bartoli et al., 2017). Mobile Al toxicity also affects the function of other plant parts, resulting in high yield reduction (Gupta et al., 2013). Breeding of alfalfa is the most promising approach to improve alfalfa production on acid soils. Variation in low soil pH and mobile Al tolerance of alfalfa plants makes it possible to breed tolerant cultivars by using different selection methods (Stevović et al., 2010; Hijbeek et al., 2021). Using adequate methods of selection and breeding during several cycles of recurrent selection have created many alfalfa varieties with great genetic yield potential and other positive traits (Petcu et al., 2006; Scott et al., 2008; Radovic et al., 2009; Khu et al., 2013; Tucak et al., 2014). There are many screening methods used to select alfalfa and other legumes adapted to mobile Al, such as: nutrient solution culture, soil bioassay, cell and tissue culture, Petri dish, hydroponic and field evaluation (Narasimhamoorthy et al., 2007; Pan et al., 2008; Khu et al., 2013). Field screening is a direct method of evaluating the tolerance to mobile Al concentrations (Fageria et al., 2009; Haling et al., 2011). However, in practice reliable ranking of accessions in the field has been difficult. Field evaluation was usually conducted in two tests, one of them in the naturally acidic soil and the other – in the soil without mobile Al. The results of these tests showed that the seed yield was significantly higher on acidic soils than under acidic soil without mobile Al concentration (Legesse and Teshale, 2020). The differences of results showed that the tolerance to mobile Al could be due to the fact that mobile Al levels were not uniform and environment factors interacted with mobile Al (Rao, 2014). The cultivars respond differently to different degrees of tolerance to mobile Al toxicity and low soil pH (Hijbeek et al., 2021). Normally, mobile Al will enter the root tip cells and inhibit root elongation, resulting in slower root growth in sensitive varieties (Panda and Matsumoto, 2007). Although Al can enter the root cells, the first toxic effect occurs due to its binding to cell walls (Rengel and Reid, 1997; Ryan et al., 1997).The plants tolerant to mobile aluminium are able to remove Al from through metabolic processes in the root system (Panda and Matsumoto, 2007). Individual plants that survive in the crop of acidic soils are usually considered the most tolerant which have shown differences in agro-biological and morphological traits. In addition, the agro-biological traits (the wintering, height of plants, stem number, seed yield) have been used to evaluate the diversity and selection of genotypes in alfalfa crops during several selection cycles (Tucak et al., 2014; Nan et al., 2019; Ta et al., 2020; Hakl et al., 2021). The yield and quantitative traits of the varieties are important part of selection and assessment in any breeding programs. The cluster analysis was used to determine the genetic diversity between the tested genotypes based on morphological and physiological traits (Moghaddam et al., 2011; Brejea et al., 2021).

The aim of the study was: 1) evaluation of tolerance of selected alfalfa genotypes using the filter-based screening methods and comparison of agro-biological traits of these alfalfa genotypes at different concentrations of mobile Al in the acidic soil; 2) estimation of cluster analysis and selection of the most promising of alfalfa genotypes based on agro-biological traits at different mobile Al concentrations.

## 2 Materials and methods

### 2.1 Plant material and research under laboratory conditions

Plant material of alfalfa was selected from the collection of the Institute of Agriculture of Lithuanian Research Centre for Agriculture and Forestry (**Table 1**). In order to evaluate and select alfalfa genotypes tolerant to acid soils, two resistance screening tests were combined under field and laboratory conditions. The collection of alfalfa genotypes sown in 2018 was evaluated in acidic soil with mobile Al concentrations (0.0–25.0 mg kg^-1^) for both growing seasons 2019–2020. After one year of testing under field conditions in 2019, the most resistant genotypes were selected, re-tested and tested for their aluminium tolerance under laboratory conditions. In 2019, the seed materials of the genotypes were used to assess resistance to mobile Al under laboratory conditions. The tolerance of alfalfa genotypes to mobile Al were assessed in different AlCl_3_ concentrations (ranged from 0.0 to 64 mM) using filter-based screening methods under laboratory conditions according to Pan et al. (2008); Buhaiov et al. (2018), and Liatukienė et al. (2020). Well-developed alfalfa seeds of similar size were scarified, the surface sterilized in the solution of 10% NaClO (sodium hypochlorite) for 30 min and rinsed three times in distilled water. The seeds were sown in Petri dishes containing two pieces of sterilized filter paper and 7 ml of sterilized 50 mM CaCl_2_ (pH 4.5) with seven concentrations of AlCl3 (aluminium chloride): 0.0, 2.0, 4.0, 8.0, 16.0, 32.0 and 64.0 mM. Thirty seeds were placed on the filter paper with three replicate dishes per treatment. The experiment was repeated twice. Petri dishes were incubated at 25°C temperature in the dark. After four days, the photoperiod was adjusted to 12/12h (day/night) at 25/20°C temperature, respectively. After three days, root and hypocotyl lengths of the seedlings were measured. The root and hypocotyl tolerance index was calculated as the maximum root and hypocotyl length in Al stress culture were divided by the root and hypocotyl length in the control treatment at 0 mM AlCl3.The genotypes of alfalfa with different tolerance to mobile Al under laboratory conditions were used in the next selection cycle in naturally acidic soil with much higher toxic concentrations of mobile Al.

**Table 1:** Cultivars and populations of alfalfa and agro-biological traits.

Number of genotype in collection	Name of genotype	Status	Origin
2	Birutė	Cultivar	Lithuania
3	Žydrūnė	Cultivar	Lithuania
4	Malvina	Cultivar	Lithuania
5	Jõgeva118	Cultivar	Estonia
6	Skriveru	Cultivar	Latvia
8	PGR12489	Cultivar	Montana, USA
13	Europe	Cultivar	France
16	AJ2024	Cultivar	Japan
17	AJC437	Cultivar	Kazakhstan
18	PGR10249	Cultivar	Nebraska, USA
19	59-109	Cultivar	Russia
28	3130	Population	Lithuania
29	3129	Population	Lithuania
30	3086	Population	Lithuania
Agro-biological traits and their description			
Wintering		Each year, the genotypes of alfalfa were evaluated at the beginning of the growing season in all plots (score 1–9, resistant crop – score 1; sensitive crop – score 9).	
Spring regrowth		The plant height of alfalfa genotypes was measured in each plot (cm).	
Height before flowering		The plant height of alfalfa genotypes was measured at the beginning of flowering (about 10.0%) in each plot (cm).	
Stem number		The number of stems was counted when the plants began flowering in each plot (m^2^).	
Seed yield		The seed yield was harvested and measured from all plots of alfalfa at the end of September (kg ha^-1^).	

### 2.2 Experimental trial in the field, meteorological conditions and statistical analysis

#### 2.2.1 Experimental trial in the field

The genotypes of alfalfa with different tolerance to mobile Al based on the filter-based screening method were sowed in the experiment field at the Vėžaičiai Branch of Lithuanian Research Centre for Agriculture and Forestry (in the western part of Lithuania, 55°70′ N, 21°49′E). The soil of the experimental site was *Bathygleyic Dystric Retisol* (WRB, 2022), with a texture of loam consisting of 8.0% clay, 45.1% silt and 46.9% sand, mobile Al (0.0–95.0 mg kg^-1^, pH 3.87–4.61). The soil was high in mobile phosphorus and mobile potassium content (177.0–335.0 mg kg^-1^ and 195.0–234.0 mg kg^-1^, respectively), N total, 0.12–0.14%.

The studies on the resistance of alfalfa genotypes of the 2020 sowing collection to mobile Al were conducted under field conditions in 2021–2022 in naturally acidic soil *Retisol*. Before the establishment of the experimental plots of alfalfa, the agrochemical characteristics of the soil were determined taking samples from a depth of 0–20 cm with a drill from each plot. The pH of soil, N, mobile P_2_O_5_ and mobile K_2_O in the soil were determined using the potentiometric, the Kjeldahl, and the Egner–Riehm–Domingo (A-L) methods, respectively. Mobile Al was determined according to the standard ISO14254:2018 (Soil quality—Determination of exchangeable acidity using barium chloride solution as an extractant).

The mobile Al concentration in the experimental site increased gradually from 0.0 to 95.0 mg kg^-1^. The lack of a homogeneous soil in terms of mobile aluminium allowed the grouping of the data, which are presented as different variations. In order to determine the effect of the causative factor, i.e. mobile aluminium, and to discover the patterns in morphological traits of alfalfa, the soil was divided into five groups according to the concentration of mobile Al: 0.0–20.0; 20.0–40.0; 40.0–50.0; 50.0–65.0 and 65.0–95.0 mg kg^-1^. The crops of alfalfa genotypes were planted on the 4^th^ of May 2020. The seeds of each alfalfa genotype were sown in two rows of 3 m length in a randomized block design with four replications. Each alfalfa cultivar and population were sown in smaller experimental plots of 1.5 m^2^ and the distance between different genotypes was 1.0 m. The agro-biological traits (the wintering, the plant height at spring regrowth and before flowering, stem number and seed yield) of alfalfa genotypes were evaluated during seasons 2021–2022 in each field of soil with different mobile Al concentrations (**Table 1**). The protection of alfalfa crops against weeds and pest was carried out using herbicide Basagran 480 (a.i. bentazon 480 g L^−1^) 2 L ha^−1^ and insecticide Mavrik 2F (a.i. tau-fluvalinate 240 g L^−1^) 0.15–0.20 L ha^−1^, respectively.

#### 2.2.2 Environment and weather characteristics

**Figure 1:**
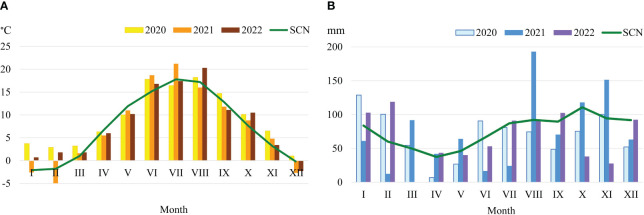
Meteorological conditions in the experimental year 2021–2022. Temperature **(A)**, Precipitation **(B)**.

Lithuania has a humid continental climate of middle latitude with snowy winters and warm summers (strong contrast between winter and summer). The western regions of Lithuania are strongly affected by the maritime climate (in winter it is warmer and in summer it is cooler than in eastern regions). The soil is more podzolized and acidic compared to other regions of Lithuania. It receives the highest rate of precipitation, which has amounted to an average of 902 mm annually during the last 10 years. Meteorological conditions in the period 2020–2022 were diverse (**Figure 1B**).

Climatic conditions were evaluated according to the data of Vėžaičiai automatic meteorological station. In 2020 and 2022, the weather was dry and warmer than usual. The amount of annual precipitation reached 92.4 and 88.4%; during the plant vegetation period – 76.3 and 87,2% of the standard climate norm (SCN). In regard to warmth, years and periods of plant growing season were 0.7–1.8°C and 0.5–0.7°C warmer compared to the SCN. The year of 2021, in regard to humidity and temperature, was favourable for the growth of perennial legume grasses (**Figure 1A**).

#### 2.2.3 Statistical analysis

The significant differences among treatment means were determined by Tukey’s test, were *p*-value was calculated, and a value of *p* < 0.05 was considered statistically significant. One-way analysis of variance (ANOVA) was used to assess the data of all agro-biological traits in each treatment of mobile Al concentrations. The interactions between the genotype × mobile Al conc., genotype × year, mobile Al conc., × year and interaction between three factors the genotype × mobile Al conc., × year in all agro-biological traits were evaluated by means of multifactor ANOVA analysis. The experimental data on all traits are presented as mean and standard error (SE) and four replicates were used for calculations. Using the hierarchical cluster analysis, it was assessed how the agro-biological traits influenced the distribution of alfalfa genotypes in different mobile Al concentrations. For statistical analyses, we used the statistical program SAS Enterprise Guide, version 7.13 (SAS Institute Inc., Cary, NC, USA).

## 3 Results

### 3.1 Evaluation of alfalfa genotypes tolerance to aluminium under the filter-based screening method

### 3.2 Evaluation of alfalfa genotype tolerance to acidic soil

**Figure 2:**
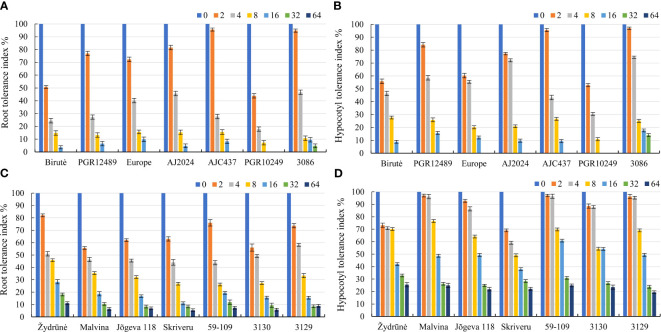
Comparision of the root and hypocotyl tolerance index of alfalfa genotypes under different concentrations of AlCl_3_ mM. **(A, B)** are moderately resistant, resistant and sensitive alfalfa genotypes, and the most resistant alfalfa genotypes are **(C, D)**. Vertical dashes indicate the mean of standard error.

The cultivar PGR10249 was the most sensitive to AlCl3 concentrations because the tolerance index of roots and hypocotyls of this genotype were the lowest at 2.0, 4.0, 8.0 mM AlCl3 concentrations (**Figures 2A, B**). The population 3086 was tolerant to 8.0, 16.0 and 32.0 mM AlCl3 concentrations. At 32 mM AlCl3, the root tolerance index of population 3086 was the highest (**Figure 2A**). The population 3086 had the best hypocotyl tolerance index at concentrations of 16.0 mM and 32.0 mM (AlCl3). However, the hypocotyl tolerance index of this population was similar to the cultivars Birute, PGR12489 and AJC437 at 8 mM AlCl3 (**Figure 2B**). The cultivars Birute, PGR12489, Europe, AJ2024 and AJC437 were medium tolerant to AlCl3. At 8.0 mM AlCl3, the root and hypocotyl tolerance index of medium tolerant genotypes ranged from 13.2% to 15.6% and from 20.1% to 27.7%, respectively. At 16.0 mM AlCl3, the root and hypocotyl tolerance index of medium tolerant genotypes ranged from 3.6% to 9.7% and from 8.8% to 15.7% (**Figures 2A, B**). The cultivars Žydrūnė, Malvina, Jõgeva 118, Skriveru, 59-109 and populations 3130 and 3129 showed the most tolerance to different concentrations of AlCl3 (**Figures 2C, D**). The root tolerance index of the most tolerant cultivars and populations was much higher compared to the cultivars Birute, PGR12489, Europe, AJ2024, AJC437, PGR10249 and population 3086 at 4.0 mM – by 15.5%, 8.0 mM – by 19.2%, 16.0 mM – by 11.8%, 32 mM – by 6.0% and 64 mM – by 7.4%. The hypocotyl tolerance index of the most tolerant cultivars and populations was also much higher compared to other cultivars and populations at 4.0 mM – by 30.2%, 8.0 mM – 42.3%, 16.0 mM – 38.4%, 32 mM – 13.6% and 64 mM – by 23.2% (**Figures 2C, D**). Evaluation of alfalfa genotype tolerance to acidic soil.

#### 3.2.1 Statistical analysis of ANOVA for agro-biological traits

The results of ANOVA showed that the effect of mobile Al concentrations was significantly large for wintering, spring regrowth, height before flowering, stem number and seed yield (**Table 2**). The effect of mobile Al showed that the field plots with different concentrations of mobile Al were contrasting and suitable to use in the selection of alfalfa genotypes for acidic soil. The effect of the year was related to climatic conditions and different concentrations of mobile Al in the soil. The effect of the genotypes showed that the genotypes varied between tolerance to mobile Al concentrations in different years. All the two-way interactions (genotype × mobile Al conc., genotype × year, and mobile Al × year) were significant for all agro-biological traits. These interactions showed that mobile Al influenced the genotypes of alfalfa during different experimental years. In addition, these interactions, in particular the interaction between the genotype × mobile Al, were significantly important for selection as the genotypes were differently affected by mobile Al concentrations. The effect of mobile Al was demonstrated by a wide range of agro-biological traits, as all the traits studied varied significantly between different concentrations of mobile Al. The interaction between genotype × mobile Al × year showed that alfalfa genotypes could be selected for tolerance to mobile aluminium on the basis of different test years and agro-biological traits.

**Table 2:** The three-way ANOVA results by the cultivar, mobile Al concentrations and year and their effect on agro-biological traits in seasons 2021–2022.

Source of Variation	Mean Squares	*P* Value	Mean Squares	*P* Value
Wintering			Spring Regrowth	
Genotype (A)	13.0214	0.0000	119.584	0.0000
Mobile Al conc. (B)	142.111	0.0000	3581.08	0.0000
Year (C)	19.1815	0.0000	7014.43	0.0000
A x B	13.9489	0.0000	94.8765	0.0000
A x C	2.3689	0.0000	178.678	0.0000
B x C	2.1433	0.0000	282.858	0.0000
A x B x C	1.4791	0.0000	126.402	0.0000
Plant Height Before Flowering			Stem Number	
Genotype (A)	960.488	0.0000	18760.2	0.0000
Mobile Al conc. (B)	24793.6	0.0000	356524.0	0.0000
Year (C)	138822.0	0.0000	225578.0	0.0000
A x B	806.622	0.0000	8561.43	0.0000
A x C	638.379	0.0000	8850.59	0.0000
B x C	3095.26	0.0000	6553.18	0.0000
A x B x C	743.751	0.0000	9710.54	0.0000
Seed Yield			Degree of Freedom in All Traits	
Genotype (A)	77048.9	0.0000	13	
Mobile Al conc. (B)	796905.0	0.0000	3	
Year (C)	293463.0	0.0000	1	
A x B	21086.0	0.0000	39	
A x C	31901.2	0.0000	13	
B x C	48191.0	0.0000	3	
A x B x C	21438.2	0.0000	39	

#### 3.2.2 The agro-biological traits of alfalfa under different mobile Al concentrations

**Figure 3:**
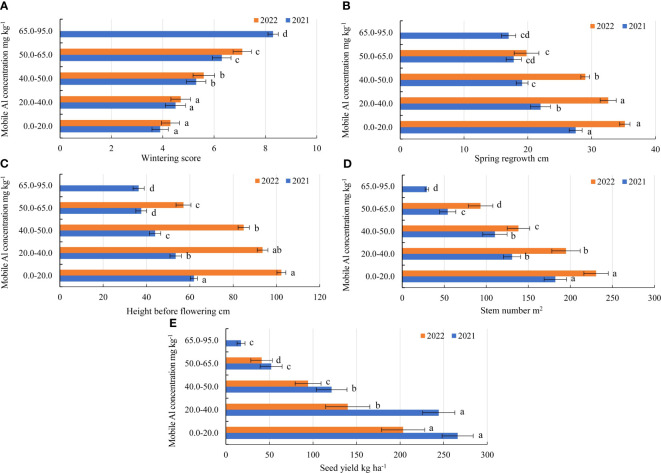
Agro-biological traits of alfalfa (14 genotypes) at different mobile Al concentrations during growing seasons of 2021–2022. **(A)** wintering; **(B)** spring regrowth; **(C)** height before flowering; **(D)** stem number; **(E)** seed yield. Vertical dashes indicate the mean of standard error.

**Figure 4:**
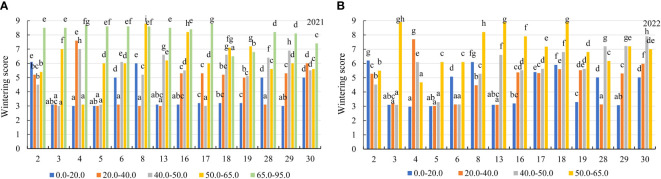
The wintering of alfalfa genotypes in the soil with different concentrations of mobile Al (mg kg^-1^) **(A)** 2021; **(B)** 2022. The differences between the cultivars with the different letters in each year are significant (*p* < 0.05). 2 – Birutė, 3 – Žydrūnė, 4 – Malvina, 5 – Jõgeva 118, 6 – Skriveru, 8 – PGR12489, 13 – Europe, 16 – AJ2024, 17 – AJC437, 18 – PGR10249, 19 – 59-109, 28 – 3130, 29 – 3129, 30 – 3086.

In the first year of use (2021), the wintering of alfalfa genotypes was similar in the soil with mobile Al concentration of 0.0–40 mg kg^-1^. The genotypes of alfalfa were more damaged by wintering in the soil at concentrations of 40.0–50.0 mg kg^-1^, 50.0–65.0 mg kg^-1^ and 65.0–95.0 mg kg^-1^ compared to mobile Al concentrations of 0.0–20.0 mg kg^-1^, by a factor of 1.4, 1.6 and 2.1, respectively (**Figure 3A**). In addition, the genotypes reacted differently to mobile Al concentrations during the winter period. In 2021, the alfalfa genotypes 3, 4, 5, 13, 16 and 29 were 2.0-fold better during wintering in the soil with a concentration of 0.0–20.0 mg kg^-1^ than the genotypes 2 and 6. At 20.0–40.0 mg kg^-1^, the wintering of the genotypes 3, 5, 6, 8, 13 and 28 were 2.5-fold better than that of genotype 4. At 40.0–50.0 mg kg^-1^, the genotypes 3, 5 and 17 were 2.7-fold lower damaged by wintering than the genotypes 4 and 29. At 50.0–65.0 mg kg^-1^, the genotype 4 was the least damaged by wintering. The genotype 18 was the best during wintering in the soil with mobile Al concentration of 65.0–95.0 mg kg^-1^ (**Figure 4A**). In the second year of use (2022), the wintering of the genotypes depended on the mobile Al concentrations in the soil and the environmental conditions during the winter period (**Figure 3A**). The wintering of the genotypes was similar in the soil with a mobile Al concentration of 0.0–40 mg kg^-1^. The genotypes were more resistant to wintering in soil with a concentration of 0.0–20.0 mg kg^-1^ compared to mobile Al concentrations of 40.0–50.0 mg kg^-1^ and 50.0–65.0 mg kg^-1^, by a factor of 1.3 and 1.6, respectively. The genotypes were the most sensitive to mobile Al concentrations of 65.0–95.0 mg kg^-1^ during winter period (**Figure 3A**)The wintering of the genotypes 3, 4, 5, 13 and 29 was 2.0-fold lower in the soil with a 0.0–20.0 mg kg^-1^ concentration compared to the genotype 2 (**Figure 4B**). At 20.0–40.0 mg kg^-1^, the genotypes 3, 5, 6, 13 and 28 were less damaged by wintering than the genotype 30 by a factor of 1.9. At 40.0–50.0 mg kg^-1^, the wintering of the genotypes 3, 5 and 6 was better than that of the genotype 30 by a factor of 2.5. At 50.0–65.0 mg kg^-1^, the genotype 4 was less damaged by wintering than the genotypes 17, 29 and 30 by a factor of 1.5 (**Figure 4B**). However, the wintering of the genotypes 3, 13 and 18 was the worst (score 8.9) because those genotypes were very sensitive to acidic soil with a mobile Al concentration of 65.0–95.0 mg kg^-1^.

**Figure 5:**
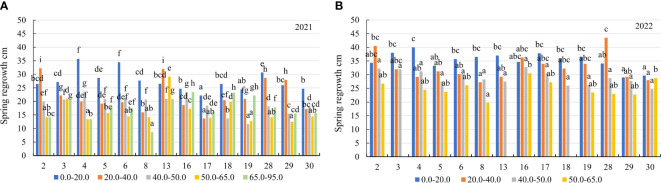
The spring regrowth of alfalfa genotypes on the soil with different mobile Al concentrations (mg kg^-1^), **(A)** 2021; **(B)** 2022. The differences between the cultivars with the different letters in each year are significant (*p* < 0.05). 2 – Birutė, 3 – Žydrūnė, 4 – Malvina, 5 – Jõgeva 118, 6 – Skriveru, 8 – PGR12489, 13 – Europe, 16 – AJ2024, 17 – AJC437, 18 – PGR10249, 19 – 59-109, 28 – 3130, 29 – 3129, 30 – 3086.

**Figure 6:**
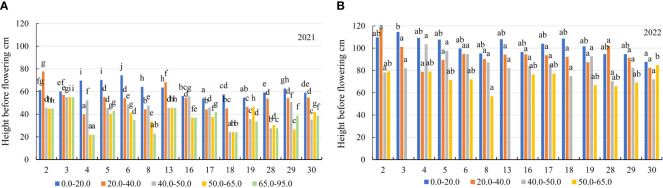
The height before flowering of alfalfa genotypes on the soil with different mobile Al concentrations (mg kg^-1^), **(A)** 2021; **(B)** 2022. The differences between the cultivars with the different letters in each year are significant (*p* < 0.05). 2 – Birutė, 3 – Žydrūnė, 4 – Malvina, 5 – Jõgeva 118, 6 – Skriveru, 8 – PGR12489, 13 – Europe, 16 – AJ2024, 17 – AJC437, 18 – PGR10249, 19 – 59–109, 28 – 3130, 29 – 3129, 30 – 3086.

In the first year of use (2021), the spring regrowth was higher on the soil with a 0.0–20.0 mg kg^-1^ concentration compared to that on the soil with 20.0–40.0 mg kg^-1^ by a factor of 1.2, and at a 40.0–50.0 mg kg^-1^ concentration – by a factor of 1.4. At 50.0–65.0 mg kg^-1^, the spring regrowth of genotypes was similar to that at a concentration of 65.0–95.0 mg kg^-1^ (**Figure 3B**). In 2021, the plant height before flowering of the genotypes also differed significantly for all mobile Al concentrations. On the soil with mobile Al 0.0–20.0 mg kg^-1^, the height before flowering was higher than that on the soil with 20.0–40.0 mg kg^-1^ by a factor of 1.2, on the soil with 40.0–50.0 mg kg^-1^ – by a factor of 1.4. The height before flowering on the soil with mobile Al 50.0–65.0 mg kg^-1^ was similar to that on the soil with a concentration of 65.0–95.0 mg kg^-1^ (**Figure 3C**). In 2021, the genotypes 4 and 6 had a 1.6-fold higher height of plants at spring regrowth than the genotype 17 in the soil with mobile Al concentration of 0.0–20.0 mg kg^-1^. At 20.0–40.0 mg kg^-1^, the spring regrowth of the genotypes 2 and 13 was higher than that of the genotype 17 by a factor of 2.3. On soil with 40.0–50.0 mg kg^-1^, the genotypes 4 and 16 had a 2.0-fold higher height of plants at spring regrowth than the genotype 19. On the soil with 50.0–65.0 mg kg^-1^, the spring regrowth of the genotype 13 was a 2.2-fold higher than that of the genotypes 4, 19 and 29. On the soil with 65.0–95.0 mg kg^-1^, the genotypes 16 and 18 had a 2.7-fold higher height of plants at spring regrowth than the genotype 8 (**Figure 5A**). In 2021, the height before flowering of alfalfa genotype 6 was (1.4-fold) higher than that of the genotype 17 on the soil with mobile Al concentration of 0.0–20.0 mg kg^-1^. On the soil with 20.0–40.0 mg kg^-1^, the genotype 2 had a 1.9-fold higher height of plants before flowering than the genotype 4. On soil with 40.0–50.0 mg kg^-1^, the height before flowering of the genotypes 3 and 16 was higher compared to the genotype 18, by a factor of 2.3. On the soil with 50.0–95.0 mg kg^-1^, the genotype 3 had a 2.5-fold higher height of the plant before flowering than the genotype 4 (**Figure 6A**).

In the second year of use (2022), the spring regrowth of genotypes was significantly similar on the soil with mobile Al 0.0–40.0 mg kg^-1^. On the soil with 0.0–40.0 mg kg^-1^, the spring regrowth was higher compared to the soil with 40.0–50.0 mg kg^-1^ by a factor of 1.2, and the soil with 50.0–65.0 mg kg^-1^ – by a factor of 1.7 (**Figure 3B**). In 2022, the height before flowering was significantly different in all mobile Al concentrations. The height before flowering was higher on the soil with a 0.0–20.0 mg kg^-1^ concentration compared to the soil with 20.0–40.0 mg kg^-1^ by a factor of 1.1, on the soil with 40.0–50.0 mg kg^-1^ – by a factor of 1.2 and on the soil with 50.0–65.0 mg kg^-1^ – by a factor of 1.8 (**Figure 3C**). In 2022, the genotype 4 had a 1.4-fold higher height of plants at spring regrowth on the soil with a concentration of 0.0–20.0 mg kg^-1^ compared to the genotypes 29 and 30. On the soil with 20.0–40.0 mg kg^-1^, the spring regrowth of the genotype 28 was (1.6-fold) higher than that of the genotypes 8 and 30. The spring regrowth of all genotypes was similar on the soil with 40.0–50.0 mg kg^-1^. On the soil with 50.0–65.0 mg kg^-1^, the genotypes 16 and 30 had a 1.5-fold higher height of plants at spring regrowth than the genotype 8 (**Figure 5B**). In 2022, the height of the genotype 3 before flowering was a 1.3-fold higher than that of the genotype 30 on the soil with a mobile Al concentration of 0.0–20.0 mg kg^-1^. On the soil with 20.0–50.0 mg kg^-1^, the height before flowering was similar in all fields of alfalfa genotypes. On the soil with 50.0–65.0 mg kg^-1^, the genotype 30 had a 1.5-fold higher height of plants before flowering than the genotype 8 (**Figure 6B**).

**Figure 7:**
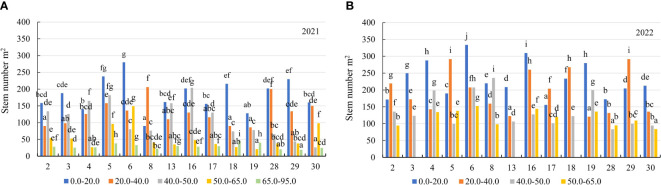
The stem number of alfalfa genotypes on the soil with different mobile Al concentrations (mg kg^-1^), **(A)** 2021; **(B)** 2022. The differences between the cultivars with the different letters in each year are significant (*p* < 0.05). 2 – Birutė, 3 – Žydrūnė, 4 – Malvina, 5 – Jõgeva 118, 6 – Skriveru, 8 – PGR12489, 13 – Europe, 16 – AJ2024, 17 – AJC437, 18 – PGR10249, 19 – 59-109, 28 – 3130, 29 – 3129, 30 – 3086.

In the first year of use (2021), the stem number of alfalfa genotypes was significantly higher in the soil with a mobile Al concentration of 0.0–20.0 mg kg^-1^ compared to the soil with a mobile Al 20.0–40.0 mg kg^-1^ by a factor of 1.4, on the soil with 40.0–50.0 mg kg^-1^ – by a factor of 1.6, on the soil with 50.0–65.0 mg kg^-1^ – by a factor of 3.3, and on the soil with 65.0–95.0 mg kg^-1^ – by a factor of 6.2 (**Figure 3D**). In 2021, the stem number of genotype 6 was a 3.1-fold higher than that of the genotype 8 in the soil with a 0.0–20.0 mg kg^-1^ concentration. In 20.0–40.0 mg kg^-1^, the genotypes 8 and 28 were higher in terms of the stem number than the genotypes 2, 18 and 19 by a factor of 2.3. On the soil with 40.0–50.0 mg kg^-1^, the stem number of the genotype 16 was 7.8-fold higher than that of the genotype 30. On the soil with 50.0–65.0 mg kg^-1^, the genotype 6 had a higher stem number than the genotype 19 by a factor of 7.1. At 65.0–95.0 mg kg^-1^, the stem number of the genotypes 5 and 19 was 2.1-fold higher than that of the genotype 29 (**Figure 7A**). In the second year of use (2022), the stem number of the genotypes was higher on the soil with concentrations of 0.0–20.0 mg kg^-1^ compared to the soil with 20.0–40.0 mg kg^-1^ by a factor of 1.2, on the soil with 40.0–50.0 mg kg^-1^ – by a factor of 1.7 and on the soil with 50.0–65.0 mg kg^-1^ – by a factor of 2.5 (**Figure 3D**). In 2022, the genotype 6 was more yielding in terms of the stem number on the soil with a concentration of 0.0–20.0 mg kg^-1^ compared to the genotype 17 by a factor of 2.1 and on the soil with 50.0–65.0 mg kg^-1^, by a factor of 1.8 compared to the genotype 30. On the soil with 20.0–40.0 mg kg^-1^, the genotypes 5 and 29 were more yielding in terms of the stem number than the genotypes 13 and 19 (2.4-fold). On the soil with 40.0–50.0 mg kg^-1^, the stem number of the genotype 8 was 2.8-fold higher than that of the genotype 28 (**Figure 7B**).

**Figure 8:**
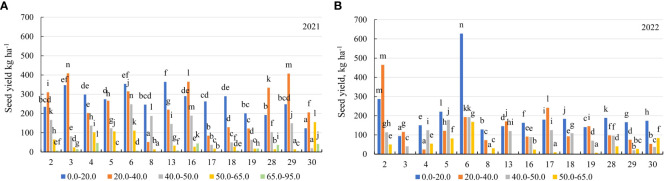
The seed yield of alfalfa genotypes on the soil with different mobile Al concentrations (mg kg^-1^), **(A)** 2021; **(B)** 2022. The differences between the cultivars with different letters in each year are significant (*p* < 0.05). 2 – Birutė, 3 – Žydrūnė, 4 – Malvina, 5 – Jõgeva 118, 6 – Skriveru, 8 – PGR12489, 13 – Europe, 16 – AJ2024, 17 – AJC437, 18 – PGR10249, 19 – 59-109, 28 – 3130, 29 – 3129, 30 – 3086.

In the first year of use (2021), the seed yield of the genotypes was similar for the soil with mobile Al concentrations of 0.0–40 mg kg^-1^ and 50–95 mg kg^-1^. For the soil with a concentration of 0.0–20.0 mg kg^-1^, the seed yield was higher than for the soil with 40.0–50.0 mg kg^-1^ by a factor of 2.1, and for the soil with 50–95 mg kg^-1^ – by a factor of 7.3 (**Figure 3E**). For the soil with a concentration of 0.0–20.0 mg kg^-1^, the genotype 13 was more yielding in terms of the seed yield than the genotype 30 by a factor of 2.9. For the soil with 20.0–40.0 mg kg^-1^, the seed yield of the genotypes 3 and 29 was 4.8-fold higher than that of the genotype 17. The genotypes 6, 30 and 4 were the most yielding in terms of the seed yield for the soil with 40.0–50.0 mg kg^-1^, the soil with 50.0–65.0 mg kg^-1^ and the soil with 65.0–95.0 mg kg^-1^, respectively (**Figure 8A**). In the second year of use (2022), the seed yield was higher with a concentration of 0.0–20.0 mg kg^-1^ compared to the soil with 20.0–40.0 mg kg^-1^ by a factor of 1.4, the soil with 40.0–50.0 mg kg^-1^ by a factor of 2.2 and the soil with 50.0–65.0 mg kg^-1^ – by a factor of 4.9 (**Figure 3E**). In 2022, the genotypes 6 and 2 were the best in terms of the seed yield for the soil with mobile Al concentrations of 0.0–20.0 mg kg^-1^ and 20.0–40.0 mg kg^-1^. The genotype 6 was the most yielding in terms of the seed yield for the soil with the concentration of mobile Al of 40.0–65 mg kg^-1^ (**Figure 8B**).

### 3.3 Cluster analysis of alfalfa agro-biological traits

**Figure 9:**
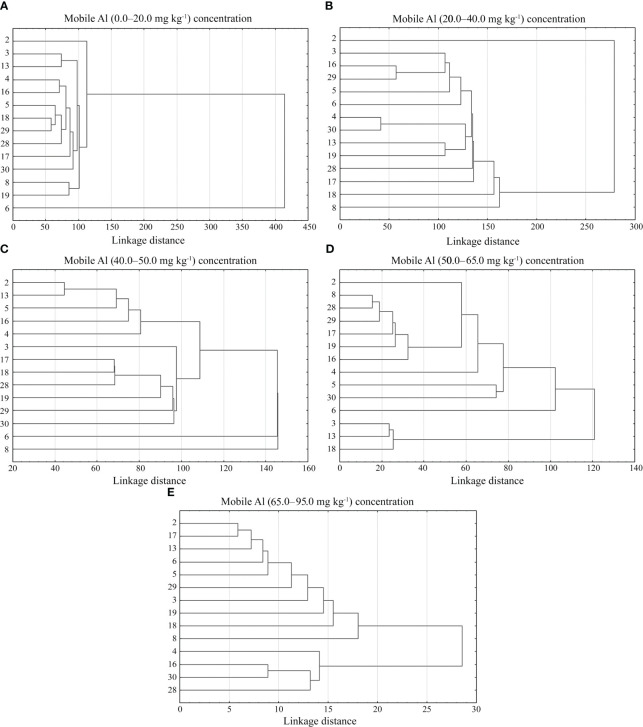
A cluster analysis of agro-biological traits on alfalfa genotypes under different mobile Al concentrations. 2 – Birutė, 3 – Žydrūnė, 4 – Malvina, 5 – Jõgeva 118, 6 – Skriveru, 8 – PGR12489, 13 – Europe, 16 – AJ2024, 17 – AJC437, 18 – PGR10249, 19 – 59-109, 28 – 3130, 29 – 3129, 30 – 3086.

The cluster analysis showed that the genotypes of alfalfa in each mobile aluminium concentration group differed in five agro-biological traits of: 0.0–20.0 mg kg^-1^, 20.0–40.0 mg kg^-1^, 40.0–50.0 mg kg^-1^, 50.0–65.0 mg kg^-1^ and 65.0–95.0 mg kg^-1^ (**Figures 9A–E**). In cluster analysis with mobile Al concentrations of 0.0–20.0 mg kg^-1^, the genotypes of alfalfa were grouped into 7 smaller clusters. The first cluster included the genotype 2. The second cluster included the genotypes 3 and 13. The third cluster included the genotypes grouped into three sub-clusters: III a (genotypes 4 and 16), III b (genotypes 5, 18 and 29) and III c (genotype 28). The genotypes of alfalfa 17 and 30 were included into the fourth and fifth clusters, respectively. The genotypes 8 and 19 were included in the sixth cluster, and the genotype 6 was included in the seventh cluster (**Figure 9A**). In cluster analysis with mobile Al concentration of 20.0–40.0 mg kg^-1^ showed the genotypes were grouped into 9 smaller clusters. The genotype 2 was included into the first cluster. The second cluster included the genotypes grouped into two sub-clusters: II a (genotype 3) and II b (genotypes 16 and 29). The genotypes 5 and 6 were included into III and IV clusters, respectively. The fifth cluster included the genotypes separated into two sub-clusters: V a (genotypes 4 and 30) and V b (genotypes 13 and 19). The genotypes 28, 17, 18 and 8 were included into the sixth, seventh, eighth and ninth clusters, respectively (**Figure 9B**). In cluster analysis with mobile Al concentration of 40.0–50.0 mg kg^-1^, the genotypes were grouped into 9 clusters. The genotypes 2 and 13 belonged to the first cluster. The genotypes 5, 16, 4 and 3 belonged to the second, third, fourth and fifth clusters, respectively. The sixth cluster contained the genotypes 17, 18 and 28, the seventh cluster contained the genotype 19, the eighth cluster contained the genotypes 29 and 30, and the ninth cluster contained the genotypes 6 and 8 (**Figure 9C**). In cluster analysis with mobile Al concentration of 50.0–65.0 mg kg^-1^, the genotypes were grouped into 7 clusters. The genotype 2 was included in the first cluster. The second cluster consisted of the genotypes 8 and 28, the third cluster consisted of the genotypes 29, 17, 19 and 16, the fourth cluster consisted of the genotype 4, the fifth cluster consisted of the genotypes 5 and 30, the sixth cluster consisted of the genotype 6, and the seventh cluster consisted of the genotypes 3, 13 and 18 (**Figure 9D**). In cluster analysis with mobile Al concentrations of 65.0–95.0 mg kg^-1^, the genotypes were grouped into 7 clusters. The genotypes 2 and 17 were included into the first cluster. The genotypes 13, 6, 5, 29, 3, 19, 18, 8 and 4 belonged to the second, third, fourth, fifth, sixth, seventh, eighth, ninth and tenth clusters, respectively. The eleventh cluster consisted of the genotypes 16 and 30, and the twelfth cluster consisted of the genotype 28 (**Figure 9E**).

## 4 Discussion

### 4.1 Evaluation of alfalfa genotype tolerance to mobile Al based on the filter-based screening method

According to authors Panda and Matsumoto (2007); Passos et al. (2012), and Hijbeek et al. (2021), the main Al damage to plant development is primarily related to cell death in the tissue of roots in direct contact with mobile Al. The root tip is the most prominent part of a plant and a sensitive organ, and it responds to micromolar Al concentrations (Huang et al., 2014; Zhu et al., 2017; Wu et al., 2022). The slower growth of the seedling aerial part occurs due to side effects related to the observed lower root development (Zhang et al., 2007). In our study, the observed differences in the behavior of genotypes when exposed to different concentrations of AlCl_3_ using the filter-based screening method clearly indicate the occurrence of genetic variability for tolerance of alfalfa genotypes and, consequently, the possibility of selection and breeding in order to obtain superior genotypes to be grown in soils with low pH and high aluminium concentrations in the soil. Based on a study of Buhaiov et al. (2018), it was found that the filter-based screening method was effective for selecting Al resistant seedlings at the juvenile plant growing stage. In addition, the results of this study showed, that the tolerance index of root and hypocotyl of all tested genotypes was 4.5% and 11.4%, respectively at AlCl_3_ concentration of 16 mM. The tolerance index of root and hypocotyl of high-tolerance genotypes ranged from 30.0% to 47.0%, and 45.0% to 100%, respectively at AlCl_3_ concentration of 7.5 mM. The tolerance index of root and hypocotyl of medium tolerant genotypes ranged from 17.0% to 33.0% and from 40% to 78.0%, respectively at AlCl_3_ 7.5 mM. Our study showed that the tolerance index of root and hypocotyl of all tested genotypes was 12.0% and 29.7%, respectively at AlCl_3_ 16 mM. In addition, the roots and hypocotyls of the medium tolerant and tolerant genotypes of alfalfa grew and developed at AlCl_3_ concentrations of 8.0 and 16.0 mM. The tolerance index of root and hypocotyl of medium tolerant and tolerant genotypes ranged from 10.7% to 15.6% and from 20.1% to 27.7%, respectively at AlCl_3_ 8 mM and from 3.6% to 9.9%, and from 8.8% to 17.7%, respectively at AlCl_3_ concentration of 16.0 mM (**Figures 2A, B**). The root and hypocotyl of very highly tolerant genotypes grew and developed at AlCl_3_ concentrations of 8.0, 16.0, 32.0 and 64.0 mM. The root tolerance index of very highly tolerant genotypes ranged from 26.2 to 45.9 at AlCl_3_ 8.0 mM, from 11.0 to 28.4 at AlCl_3_ 16.0 mM, from 8.4 to 18.1 at AlCl_3_ 32.0 mM, and from 5.4 to 11.1 at AlCl_3_ 64.0 mM. The hypocotyl tolerance index of very highly tolerant genotypes ranged from 49.1 to 70.2 at AlCl_3_ 8.0 mM, from 37.9 to 60.8 at AlCl_3_ 16.0 mM, from 23.8 to 32.9 at AlCl_3_ 32.0 mM, and 19.5 to 25.5 at 64 AlCl_3_ mM (**Figures 2C, D**). Our study showed that the filter-based screening method can be used to select promising genotypes of alfalfa. Furthermore, this method can be successfully applied for repetitive selection to select genotypes tolerant to acidic soil under field conditions and re-screened under laboratory conditions over several selection cycles (Barone et al., 2008; Pan et al., 2008; Buhaiov et al., 2018).

### 4.2 Agro-biological traits of alfalfa on acidic soil with different mobile Al concentrations

Wintering survival of alfalfa genotypes under the field conditions depended on how it well-established during the year of sowing (Djaman et al., 2021). The most important factor, which shows successful wintering survival of alfalfa crops is a well-developed root system (Xu et al., 2020; Xu et al., 2020b). Our study showed that the wintering of the alfalfa genotypes was better in the soil with mobile Al concentration of 0.0–40.0 mg kg^-1^ in 2021. During the winter period, the weather conditions were not so critical for the wintering of alfalfa genotypes. However, the genotypes of alfalfa suffered more after wintering and the crops of alfalfa were more thinned in the soil with mobile Al 40.0–95.0 mg kg^-1^ in 2021. In 2022, the weather conditions were also not critical for wintering of alfalfa. In the soil with mobile Al concentration of 40.0–95.0 mg kg^-1^, the wintering of alfalfa crop was more affected by thinning or death of alfalfa plants during the winter period (**Figure 3A**). Quick regrowth in spring is a very important factor, as the alfalfa canopy is closely linked to the morphological structure of the stems and well-developed roots, and the roots determine the yield and quality of alfalfa (Bélanger et al., 2006; Xu et al., 2020). Root elongation is inhibited under water stress and excess Al, and thus affects the growth and development of stems (Yang et al., 2009). The plant response to drought stress is the inhibition of shoot growth due to low soil moisture. Water deficit causes a complex response characterised by a reduction in the water potential of plant tissues, leading to modifications in various plant processes (Blum, 2010; Rosales et al., 2012). Our studies showed that the alfalfa genotypes were subjected to drought and mobile Al stress on the plant height at spring regrowth, before flowering and stem number due to warm and dry weather conditions during the period of spring–summer in 2021. In 2021, the spring regrowth and height before flowering were lower than in 2022 by a factor of 1.3 and by a factor 1.6, respectively at a concentration of 0.0–20.0 mg kg^-1^, by a factor of 1.5 and 1.7, respectively at a concentration of 20.0–40.0 mg kg^-1^, by a factor of 1.5 and by a factor of 1.9, respectively at a concentration of 40.0–50.0 mg kg^-1^, and by a factor of 1.1 and 1.5, respectively at a concentration of 50.0–65.0 mg kg^-1^. (**Figures 3B, C**). The stem number was lower in 2021 compared to 2022 by a factor of 1.3 at a concentration of 0.0–20.0 mg kg^-1^, by a factor of 1.5-fold at a concentration of 20.0–40.0 mg kg^-1^, by a factor of 1.3-fold at a concentration of 40.0–50.0 mg kg^-1^, and a factor of 1.7 at a concentration of 50.0–65.0 mg kg^-1^. (**Figure 3D**). Our study showed that the genotypes of alfalfa also differed by the plant height at spring regrowth and before flowering under different environmental conditions during the growing seasons in 2021–2022. Plant height is related to the environmental conditions and the genetics of individual genotypes (Kavut et al., 2014; Davodi et al., 2011; Djaman et al., 2020). Sunny, warm weather with low rainfall is favourable for alfalfa seed yields, as these environmental conditions result in a long flowering period for alfalfa, which is favourable for bee pollination. Seed yield will be related to the density of the crop, which varies between alfalfa genotypes Morante and Lira, 2018; Hossain et al., 2020; Inal, 2023). Our study showed that alfalfa seed yields were significantly higher in the years with low rainfall compared to rainy years in the soil with different mobile Al concentrations. The seed yield of the genotypes was higher in 2021 compared to 2022 by a factor of 1.3 at a concentration of 0.0–20.0 mg kg^-1^, by a factor of 1.7 at a concentration of 20.0–40.0 mg kg^-1^, and by a factor of 1.3 at a concentration of 40.0–65.0 mg kg^-1^ (**Figure 3E**).

Cluster analysis is an effective tool for determining the degree of genetic variability between the genotypes under study in terms of their performance and contributing traits (Qiang et al., 2015). Several studies have shown that in any breeding programme, it is important to select and evaluate cultivars for quantitative and yield characteristics so that the cultivars can be introduced in a given local environment (Brejea et al., 2021). Various authors identified different cluster analysis groups of legume grasses based on the contribution of morpho-agronomic traits. According to Tucak et al. (2009); Asci (2011), and Petrović et al. (2014), different red clover genotypes were grouped into eight, six and three clusters, respectively, on the basis of agro-biological traits, such as plant height, number of stems, seed yield, and green and dry matter yields. In addition, these authors found the most promising traits of red clover and selected populations that could be of interest of breeding. Touil et al. (2009) grouped 35 alfalfa populations into three clusters based on nine morphological traits, which differed significantly in terms of morphological traits. Riasat et al. (2021) classified 51 alfalfa genotypes into four cluster groups based on forage yield, plant height and forage quality traits. Our study showed that the genotypes of alfalfa differed for 5 agro-biological traits in each cluster with different mobile Al concentrations (**Figure 9**). The selected genotypes with valuable traits will be used in breeding for the development of the cultivars tolerant to acidic soils. In cluster analysis with mobile Al (0.0–20.0 mg kg^-1^) concentrations, the most tolerant genotypes (3, 4, 5, 6, 19, 28, 29) to aluminium under laboratory conditions were distributed in different clusters. The genotype 3 in cluster II and the genotype 4 in sub-cluster III-a were the most tolerant in terms of wintering. The genotype 3 in cluster II and the genotype 28 in sub-cluster III-c had a similar plant height at spring regrowth. The genotype 4 in sub-cluster III-a and the genotypes 5 and 29 in III-b, were similar by the plant height before flowering. In sub-cluster III-c and cluster V, the genotypes 28 and 30 were similar in terms of the stem number. The genotype 3 in cluster II and the genotype 5 and 29 in sub-cluster III-b were similar in seed yield. In cluster VII, the genotype 6 had the best values of the spring regrowth, height before flowering, stem number and seed yield (**Figure 9A**). In cluster analysis with mobile Al (20.0–40.0 mg kg^-1^) concentrations, the most tolerant genotypes under laboratory conditions were included into different clusters. In sub-cluster II-a, the genotype 3 had similar height before flowering compared to the genotype 28 in cluster VI and the stem number to the genotypes 4 and 30 in sub-cluster V-a. In sub-cluster II-b, the genotype 29 had a similar height before flowering compared to the genotype 6 in cluster IV and the genotype 19 in sub-cluster V-b. In cluster III, the genotype 5 was the best in terms of wintering and stem number. In cluster IV, the genotype 6 was similar in terms of wintering and spring regrowth to the genotype 5 in cluster III, and in terms of height before flowering to the genotypes 29 and 19 in sub-clusters II-b and V-b, respectively. In sub-cluster V-a, the genotypes 4 and 30 were similar in terms of spring regrowth and stem number compared to the genotype 3 in sub-cluster II-a, respectively. The genotype 28 in cluster VI had the best height at spring regrowth and had similar wintering properties compared to the genotype 5 in cluster III and the genotype 6 in cluster IV (**Figure 9B**). In cluster analysis with mobile Al (40.0–50.0 mg kg^-1^) concentrations, the most tolerant genotypes to aluminium under laboratory conditions were divided into different smaller clusters. In cluster II, the genotype 5 was similar in wintering to the genotype 3 in cluster V, in spring regrowth to the genotype 28 in cluster VI and in stem number to the genotype 19 in cluster VII. In cluster IV, the genotype 4 had the best value of the height before flowering and stem number. In cluster V, the genotype 3 was the best in wintering. In cluster VIII, the genotypes 29 and 30 were sensitive to wintering, and had the lowest value of the stem number and seed yield. In cluster IX, the genotype 6 was the best in seed yield (**Figure 9C**). In cluster analysis with mobile Al (50.0–65.0 mg kg^-1^) concentrations, the most tolerant genotypes to aluminium under laboratory conditions were divided into smaller clusters. In cluster II, the genotype 28 had similar seed yield to the genotype 3 in cluster VII. In cluster III, the genotype 29 had similar height before flowering and stem number to the genotype 4 in cluster IV. In cluster IV, the genotype 4 was the best for wintering. In cluster V the genotypes 5 and 30 had similar spring regrowth to the genotype 6 in cluster VI. In cluster VI, the genotype 6 had the best value of the height before flowering, stem number and seed yield. In cluster VII, the genotype 3 was the best in spring regrowth (**Figure 9D**). In cluster analysis with mobile Al (65.0–95.0 mg kg^-1^) concentrations, the most tolerant genotypes to aluminium under laboratory conditions were divided into smaller clusters. In this cluster analysis, the genotypes had the lowest value for agronomic traits, but individual genotypes differed for the best traits. The values of the agro-biological traits allowed the selection of genotypes, albeit from single plants, which may be valuable for breeding. In cluster VI, the genotype 3 was similar to the genotype 13 in cluster II in spring regrowth. In cluster III, spring regrowth, height before flowering and seed yield of the genotype 6 were similar to the genotypes 28 and 19 in clusters XII and VII, respectively. In cluster IV, the stem number of the genotype 5 was similar to the genotype 19 in cluster VII. In cluster VI, the genotype 3 had the best value of the height before flowering. In cluster X, the genotype 4 had the best value of the seed yield. In cluster XI, the genotypes 30 were similar in stem number to the genotype 4 in cluster X (**Figure 9E**).

## 5 Conclusions

The results of this study revealed a wide phenotypic diversity of fourteen genotypes of alfalfa for the traits studied in the laboratory and in naturally acidic soil. The study results showed that the diversity of agro-biological traits significantly depended on mobile Al concentrations. Cluster analysis with mobile Al concentrations of 0.0–65.0 mg kg^-1^ showed that, the genotypes Žydrūnė, Malvina, Europe, AJ2024, Jõgeva 118, and 3130 had the best value for wintering, the genotypes 3130, Skriveru, Birutė, AJ2024, Žydrūnė and Europe had the best value for spring regrowth. In addition, the height before flowering of the genotypes Skriveru, Birutė, Malvina was the best, the seed yield of the genotypes Skriveru, Birutė, PGR12489 was the best and the genotypes Skriveru, Jogeva118, and Malvina had the best value for stem number.

The filter-based screening method revealed that the genotypes Žydrūnė Malvina, Jõgeva 118, Skriveru, and 3130 were the most tolerant, the hypocotyl tolerance index of these genotypes was higher compared to medium tolerant genotypes of alfalfa at AlCl_3_ concentrations 8, 16, 32 and 64 mM. The genotypes Birutė, PGR12489, Europe and AJ2024 were medium tolerant to AlCl_3_ concentrations when using the filter-based screening method. The hypocotyl and root tolerance index of these genotypes was higher compared to sensitive genotype PGR10249 at 8 and 16 mM AlCl_3_. The genotypes of alfalfa 59-109, 3129, 3086 and PGR 10249 differed in tolerance to AlCl_3_ concentrations when using the filter-based screening method. However, cluster analysis showed that the genotypes 59-109, 3129 and 3086 varied in agro-biological traits in the soil with all mobile Al concentrations, however, they did not exhibit any distinctive traits compared to other genotypes.

Generally, this study of the first and second year of use (2021–2022) showed that the genotypes of alfalfa grew and developed in the soil with mobile Al concentration of 0.0–40 mg kg-1. At this concentration, the genotypes of alfalfa had significantly better values of agro-biological traits compared to mobile Al concentrations of 40.0 to 95.0 mg kg-1.

## Data availability statement

The original contributions presented in the study are included in the article/supplementary material. Further inquiries can be directed to the corresponding author.

## Author contributions

AL: Conceptualization, Data curation, Formal analysis, Methodology, Resources, Software, Supervision, Validation, Visualization, Writing – original draft. RS: Conceptualization, Data curation, Formal analysis, Investigation, Methodology, Resources, Supervision, Validation, Visualization, Writing – original draft. EN: Funding acquisition, Writing – review & editing. ST: Writing – review & editing. PP: Writing – review & editing. GP: Software, Writing – original draft.
